# Commentary on: Toward Successful International Pooling of Breast Implant Registry Data: The Role of Dataset Uniformity

**DOI:** 10.1093/asjof/ojag120

**Published:** 2026-06-17

**Authors:** Caroline Glicksman

See the Original Article here.

Science knows no country, because knowledge belongs to humanity, and is the torch which illuminates the world.—Louis Pasteur

The International Collaboration of Breast Registry Activities (ICOBRA), a suborganization of the International Confederation of Plastic Surgery Societies, was established in 2013, with an ambitious goal to create a common language for breast implant surveillance across registries that were developed independently and at different time points. The need for such a collaboration became apparent following the Poly Implant Prothèse crisis and the global emergence of breast implant–associated large cell lymphoma, both of which highlighted the limitations of isolated international databases in detecting rare but clinically significant implant-related events. Since its inception, ICOBRA has grown to include 9 international breast implant registries. Contributing registries now include Australia, the Netherlands, Germany, France, Sweden, Italy, and 2 from the United States, the American Registry for Breast Implant Surveillance (ARISE) and the National Breast Implant Registry (NBIR).

The authors are to be congratulated for accomplishing something far more challenging than establishing a registry: they created a framework that allows registries developed independently, under different regulatory systems and healthcare environments, to communicate.^1^ Differences in definitions, classification systems, procedural coding, and registry objectives can profoundly affect pooled analysis. Over the last several years, ICOBRA has worked systematically to make diverse datasets comparable and to align data from previously unlinked registries. The group encountered significant differences in registry design, capture rates, procedural and device descriptions, and, of course, language.^2,3^

An important lesson from this paper is that harmonization is not a one-time accomplishment but a continuous process. The authors describe the process of harmonizing data across registries that differ not only in platform design but also in the specific national interests of surgeons, patients, and regulators in each country. The revised ICOBRA dataset addresses these challenges by refining surgical indication categories, clarifying definitions for primary and revisional procedures, and standardizing capture of implant-related complications. Data now includes more details on implant explantation and the smaller subset of patients who elect to replace their implants after a previous explantation. In addition, several terms were modified or consolidated into a single category, including autoimmune syndrome induced by adjuvants, breast implant illness, and systemic symptoms associated with implants. The revised dataset harmonized these terms to capture patient-reported systemic symptoms without presuming causation and promotes consistent reporting across registries.

Not all implants are approved or commercially available in every country, and even within a single manufacturer's portfolio, substantial variation may exist in gel formulations, implant shapes, and device generations. Currently, 4 silicone gel implant manufacturers and 1 saline implant manufacturer market and distribute implants in the United States. The 4 silicone implant manufacturers are integrated with ARISE through unique device identification technology, enabling immediate device registration. In addition, ICOBRA has updated implant characteristics, including implant surface classification that aligns with the 2024 International Organization for Standardization (ISO) classification standards.^4^ ICOBRA classifies implants based on surface characteristics, identifying smooth (<10 µm), microtextured (10-20 µm), and macrotextured (>20 µm) surfaces. However, debate is continuing as to the classification of certain implant surfaces that, according to the new ISO classification, have a manipulated surface and are, therefore, classified as microtextured.

Data protection within countries, and especially in the context of cross-border data sharing, presents significant challenges. ICOBRA has established a framework that ensures participating registries share only fully anonymized, aggregate-level data internationally. In 2026, 2 additional registries joined ICOBRA: the French Registry and ARISE. Leveraging processes established and refined over recent years, the ARISE developers were provided with a comprehensive implementation framework, including detailed step-by-step guidance to align collected data using the recently updated ICOBRA core dataset definitions and data dictionary. Although ARISE was not directly involved in the modified Delphi consensus process, there appears to be strong concordance between ARISE data elements and the established ICOBRA standards across the majority of variables.

Dataset harmonization is only as valuable as the completeness of the data. In several participating countries, registries were developed under a clear regulatory mandate. In the United States, both the NBIR and ARISE satisfy this requirement, but an important issue remains unaddressed. Australia, the Netherlands, Sweden, Italy, and Germany have implemented mandatory or “opt-out” breast implant registries. In contrast, participation in the United States remains voluntary. Although both ARISE and NBIR have made substantial contributions, together they capture only a fraction of implants placed annually in the United States. High-quality surveillance requires not only standardized datasets but also across-the-board participation. Without adequate capture rates, even the most comprehensive registry platform cannot fully realize its potential. A recent publication by Chow et al derived data from the Australian Breast Implant Registry over a 6-year period. They looked at procedural data for primary augmentation and the subsequent development of capsular contracture.^5^ This study was made possible by the exceptionally high level of registry coverage in Australia, where ∼98% of devices sold are captured within the national registry. These high capture rates pave the way for registries to provide a safer and less expensive means for manufacturers to bring new devices to market, while also delivering postapproval safety data more effectively than current regulations allow.^6^ A major limitation in the United States is the absence of manufacturer-driven or regulatory mandates requiring that all implants sold be registered within 1 of the 2 national registries.

Because breast implant registries continue to mature, the next challenge is not just harmonization; it is achieving the level of participation and data completeness to transform surveillance data into actionable evidence.
